# Effects of Hyperbaric Oxygen Combined with Lycopene on Oxidative Stress and Inflammation in Collegiate Badminton Players After High-Intensity Training

**DOI:** 10.3390/nu18172861

**Published:** 2026-09-02

**Authors:** Yang Xiang, Zihan Peng, Beilun Fu, Youling Qian, Tao Ma, Binghong Gao, Huan Zhu

**Affiliations:** 1 Faculty of Health Sciences and Physical Education, Macao Polytechnic University, Macao, China; xiangyang526526@163.com; 2 School of Sports, Hubei Minzu University, Enshi 445000, China; 3 Intelligent Technology of Selenium Food Nutrition and Health, Hubei Engineering Research Center, Hubei Minzu University, Enshi 445000, China; 4 Faculty of Sports, Ningbo University, Ningbo 315211, China

**Keywords:** hyperbaric oxygen recovery, lycopene, high-intensity exercise, oxidative stress, inflammation, exercise fatigue

## Abstract

**Objectives:** This study aims to compare the effects of three interventions, namely hyperbaric oxygen (HBO) therapy, HBO combined with lycopene (LYC) supplementation, and natural recovery, on oxidative stress and inflammatory responses following high-intensity exercise. The goal is to provide a theoretical foundation and methodological reference for strategies that help reduce fatigue after intense physical activity. **Methods:** Collegiate badminton players (*n* = 45) were randomly assigned to control, HBO, and HBO+LYC groups. Each group completed a 4-week intervention in accordance with its assigned protocol. The levels of oxidative stress, inflammation-related markers, and exercise-induced fatigue indicators were assessed before and after the intervention. Changes in these markers following high-intensity exercise were analyzed using repeated-measures ANOVA to evaluate the effects of HBO and HBO+LYC. **Results:** Following the intervention, the control group showed significant increases in malondialdehyde (MDA), interleukin-6 (IL-6), tumor necrosis factor (TNF), C-reactive protein (CRP), creatine kinase (CK), blood urea (BU), and heart rate (HR), along with a significant decrease in testosterone (T) (all *p* < 0.05). Compared with the control group, both intervention groups had significantly lower MDA, IL-6, TNF, CRP, CK, BU, and HR and higher superoxide dismutase (SOD), catalase (CAT), and total antioxidant capacity (T-AOC) (all *p* < 0.05). Compared with the HBO group, the HBO+LYC group showed significantly higher CAT and lower IL-6 and CK levels (all *p* < 0.05). **Conclusions:** Both HBO alone and HBO+LYC effectively reduced oxidative stress, inflammation, and exercise-induced fatigue, as evidenced by decreased MDA, IL-6, TNF, CRP, CK, BU, and HR, along with increased SOD and CAT, T-AOC, and T. Moreover, HBO+LYC demonstrated superior efficacy over HBO alone for selected outcomes, particularly by further enhancing glutathione peroxidase 3 activity. These findings suggest that HBO, especially when combined with LYC, may be an effective strategy to attenuate oxidative stress and inflammation and promote recovery following high-intensity exercise.

## 1. Introduction

Badminton is a fast-paced racket sport with high participation rates and widespread popularity among the general population in many countries, particularly in Asian countries such as China [1,2,3]. During competition, athletes perform rapid and frequent high-intensity movements, including changes in direction, abrupt stops, and jumps, which impose substantial exercise loads. These high-intensity workloads can induce acute cellular hypoxia, which promotes free radical production and triggers oxidative stress. The resulting oxidative stress may further induce inflammatory responses and adversely affect athletes’ health [4,5,6]. Following high-intensity exercise, exercise-induced hypoxia promotes excessive free radical production, disrupting the balance between pro-oxidant and antioxidant systems, accelerating lipid peroxidation, and ultimately resulting in oxidative damage to cells [7]. Oxidative stress activates the NF-κB-signaling pathway, which in turn promotes the release of pro-inflammatory cytokines and amplifies systemic inflammation [8]. Concurrently, neuroinflammation stimulates glial cells to generate reactive oxygen species (ROS) and reactive nitrogen species (RNS), further intensifying oxidative stress. This self-perpetuating cycle leads to mitochondrial dysfunction and impaired muscle cell contraction, ultimately contributing to the development of exercise-induced skeletal muscle fatigue [9]. Failure to effectively mitigate oxidative stress and inflammation in badminton players after exercise can lead to cumulative cellular damage and persistent inflammation, ultimately impairing training quality, reducing athletic performance, and increasing the risk of sport-related injuries [10,11]. Existing recovery strategies, such as physical massage and dietary supplementation, may help alleviate post-exercise oxidative stress but remain largely non-specific and do not directly address cellular hypoxia [12,13]. Consequently, the development of safe and targeted interventions that specifically reduce exercise-induced cellular hypoxia remains an important challenge in sports science.

Hyperbaric oxygen (HBO) therapy involves placing the body in an environment where atmospheric pressure exceeds one atmosphere absolute (ATA) while breathing high concentrations of oxygen. This treatment enhances the solubility and partial pressure of oxygen in the blood, increases the oxygen gradient between blood and cells, extends the effective diffusion distance of oxygen, and ultimately improves tissue oxygen delivery [14]. Owing to its demonstrated effectiveness, HBO has increasingly been incorporated into athletic training protocols as an evidence-based strategy to counteract exercise-related fatigue [15,16]. However, in cases of profound fatigue or high-intensity exercise that induces marked elevations in oxidative stress, HBO therapy alone may be insufficient to fully neutralize free radicals, allowing residual oxidative damage to persist after treatment. Evidence from a randomized crossover trial demonstrated that a 60 min HBO session at 1.3 ATA following high-intensity exercise alleviated mood disturbances in participants but did not significantly reduce oxidative stress markers, decrease reactive oxygen species (ROS) derivatives, or enhance bio-antioxidant potential [17]. Similar findings were reported by Park et al. [18]. In their study, a 30 min HBO session at 1.3 ATA following high-intensity exercise effectively lowered participants’ heart rate and blood lactate levels but had no significant effect on antioxidant capacity.

Lycopene (LYC), a carotenoid pigment present in red and pink fruits and vegetables, is widely recognized for its strong antioxidant properties. It has been shown to reduce post-exercise lipid peroxidation and promote muscle recovery following high-intensity exercise [19,20]. Di et al. reported that LYC supplementation significantly enhanced an tioxidant nonproteic capacity and decreased serum lipid hydroperoxides levels in subjects after exercise [21]. Similar results were reported by Yu et al. [22]. In animal experiments, the authors found that LYC reduced malondialdehyde (MDA) levels and Increasing glutathione peroxidase 3 (GPX3) activity in mice thereby effectively reduced oxidative stress damage. Thus, both HBO and LYC alone can reduce oxidative stress and inflammatory responses following high-intensity exercise. Accordingly, it was hypothesized that combining the two may produce an additive effect, thereby more effectively scavenging free radicals in the body. However, if used together, they may also produce an antagonistic effect, which could weaken or offset the intervention’s efficacy. However, no scholars have yet been found to have conducted targeted research on the above hypothesis. Based on this background, to clarify the effects of a combined intervention of the two on oxidative stress and inflammatory responses following high-intensity exercise, this study focuses on badminton players, combined the two interventions and compared their efficacy with that of HBO therapy alone so as to determine a more effective method for alleviating exercise-induced fatigue after high-intensity exercise.

## 2. Methods

### 2.1. Participants

This study recruited 45 badminton player student athletes from the School of Physical Education at Hubei Minzu University (Enshi, China) as participants. All individuals participated voluntarily and provided their written informed consent. The study protocol was approved by the Ethics Committee of Hubei Minzu University for Scientific Research and Biomedical Ethics (approval number: 2024072 and date of 14 September 2024). This study strictly adhered to the principles of the Declaration of Helsinki and received formal approval (Approval No.: ChiCTR2500109232 and date of 15 September 2025) from the Chinese Clinical Trial Registry.

#### 2.1.1. Inclusion Criteria

The inclusion criteria were as follows: (1) Student athletes specializing in badminton at the School of Physical Education of Hubei Minzu University, with the time of moderate-to-high-intensity exercise (≥70% of maximum heart rate, maximal incremental exercise test to determine maximum heart rate [23]) ranging from 14 to 18 h per week. (2) Demonstration of normal cognitive and communication competence. (3) Physical and mental health, good-quality sleep, no mental anxiety, nervousness, or claustrophobia. (4) Voluntary agreement to participate in the full trial protocol after undertaking a comprehensive understanding of the study procedures, with written informed consent obtained.

#### 2.1.2. Exclusion Criteria

The exclusion criteria were as follows: (1) Participants who were unable to ensure daily completion of the intervention tasks. (2) Participants engaged in moderate-to-vigorous physical activity for <14 h or >18 h per week. (3) After professional testing in the hospital, those with absolute or relative contraindications to HBO or an allergy to LYC [24,25]. (4) Individuals who had taken antioxidants or drugs that may affect the absorption of LYC (such as vitamin C, vitamin E, and astaxanthin) within the past month. (5) Participants with recent (within the past month) injuries that could affect normal training or those who had participated in other intervention trials. (6) Other participants were deemed unsuitable for their participation in this study.

#### 2.1.3. Sample Size Estimation

**Figure 1:**
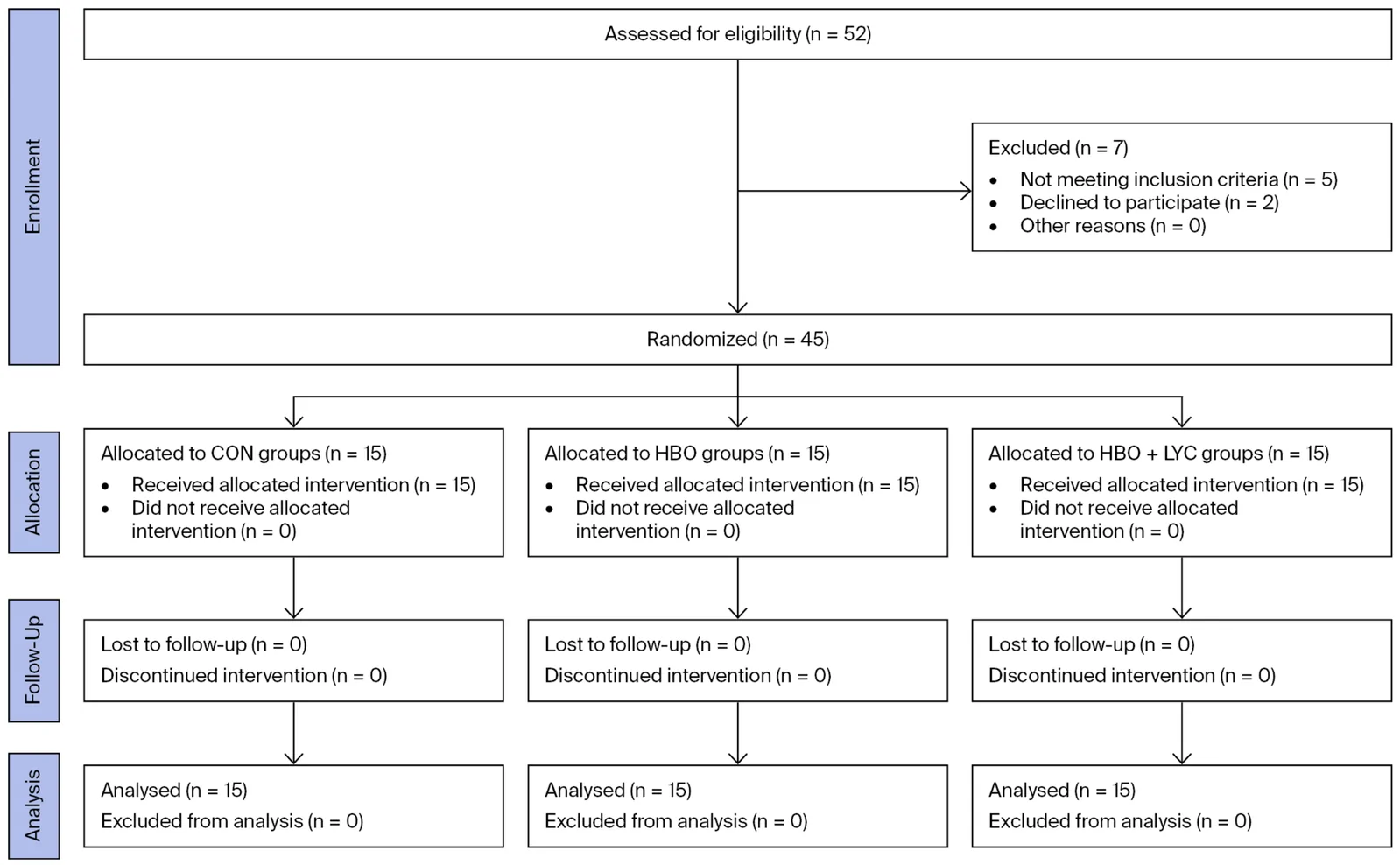
CONSORT Flow Diagram.

The sample size was estimated using the G*Power 3.1 software (v.3.1.9.2 Heinrich-Heine-Universität Düsseldorf, Düsseldorf, Germany), setting the effect size in accordance with the calculation of Cohen’s guidelines with a significance level of α = 0.05, a power of 1 − β = 0.80, and an effect size of 0.3 [26]. The estimated total sample size was 30 participants. To account for potential attrition, the sample size was increased to 45 collegiate badminton players, with 15 participants in each group (10 males and 5 females). Using a stratified cluster-randomized design, the athletes were first categorized into male and female groups. Next, based on their baseline MDA levels, the participants were further assigned to subgroups of 3 participants each. Finally, the men athletes were assigned to 10 subgroups and women to 5 subgroups. Subsequently, computer-simulated grouping was used to randomly assign the paired athletes to the CON, HBO, and HBO+LYC groups (all *n* = 15) (Figure 1). Computer-generated randomization was then performed to allocate the matched athletes into three groups: control (CON), HBO and HBO+LYC. The participants were tested using the InBody770 Body Composition Analyzer (InBody Co., Ltd., Seoul, Republic of Korea) at 08:00 a.m. and instructed to refrain from eating before the test, to keep their palms and soles dry, to place their feet on the electrode plates, hold the handles, and maintain a 45° angle between their upper limbs and trunk. These measurements included body weight and basal metabolic rate, among others (Table 1).

### 2.2. Test Method

#### 2.2.1. Training Schedule

##### Control of Exercise Volume and Intensity

Exercise intensity and volume were monitored with reference to the Rating of Perceived Exertion (RPE) scale (CR-10), blood lactate levels, and heart rate monitors during specialized physical training sessions. In addition, session-RPE (s-RPE) was employed to assess the exercise intensity and volume during both specialized physical training and specialized technical training sessions [27,28] (Table 2).

#### 2.2.2. Treatment Protocol

##### HBO-Intervention Protocol

**Figure 2:**
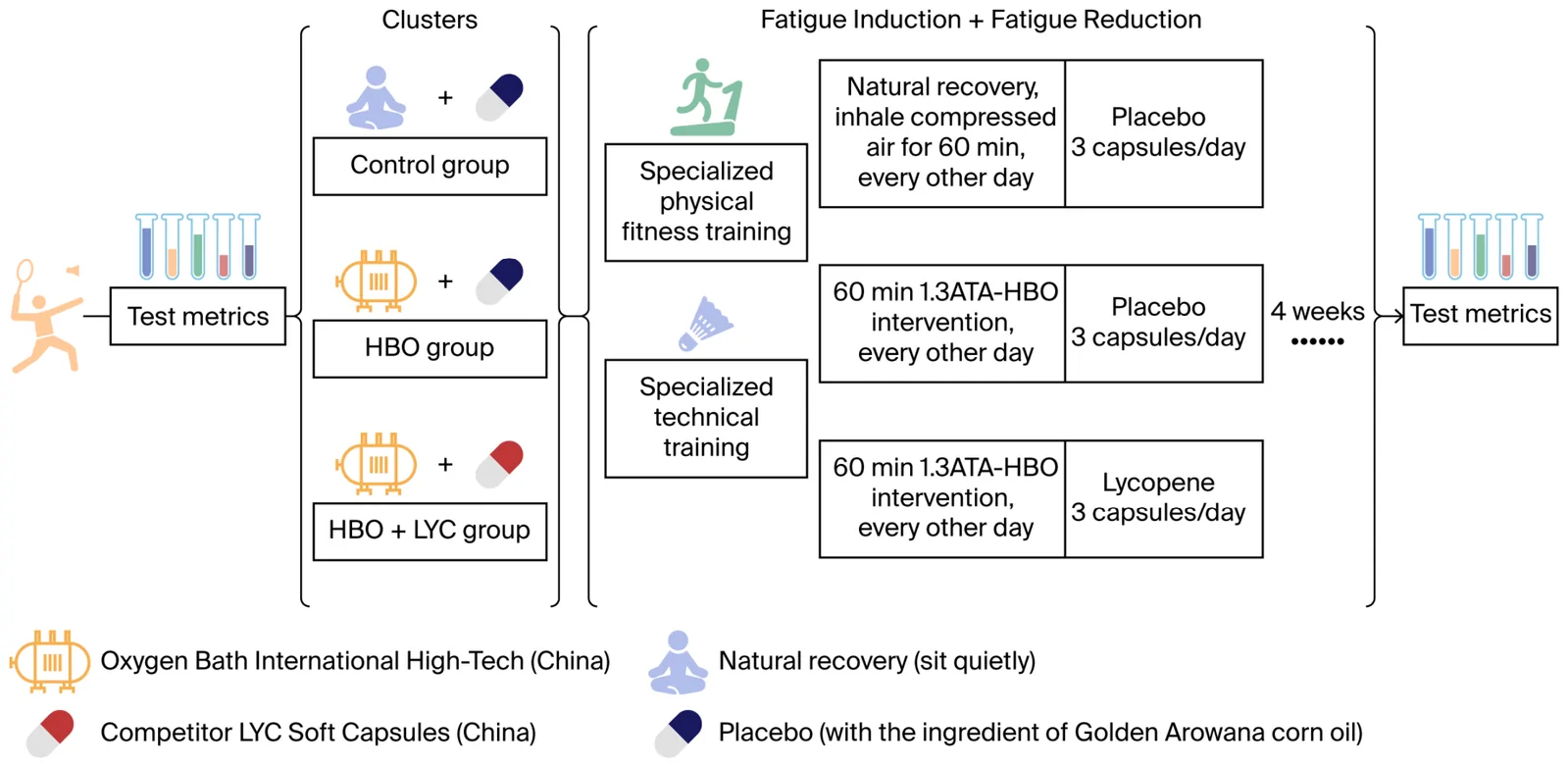
Flowchart illustrating the experimental process.

Current evidence indicates that a course of 60 min HBO therapy sessions over 4 consecutive weeks was effective in reducing oxidative stress damage and in accelerating recovery from exercise-induced fatigue [15,16]. On this basis, the subjects of both the HBO and HBO+LYC groups received a 60 min HBO therapy session every alternate day for 4 weeks. The chamber pressure was maintained at 1.3 ATA with an oxygen concentration of 26–28%. The participants lay supine in the chamber, followed by a 5 min compression phase to reach 1.3 ATA. Steady-state oxygen inhalation was maintained for 60 min under 1.3 ATA. The session was concluded by gradual decompression to 1 ATA over 5 min (Figure 2). All procedures were supervised by certified technicians who continuously monitored the participants for any adverse reactions. The participants were instructed to report any discomfort immediately; manual pressure relief valves were available for emergency decompression. Pre-trial Adaptation: Habituation phase: 2–3 simulated sessions were conducted to familiarize the participants with HBO procedures, including the potential side-effects (e.g., ear pain and tinnitus) and mitigation strategies (e.g., mouth opening or saliva swallowing). Washout period: A 7-day interval was implemented after habituation to eliminate any residual adaptation effects before formal experimentation.

##### LYC-Intervention Protocol

Subjects in the HBO+LYC group received LYC supplementation in addition to a 4-week HBO therapy regimen (60 min per session, administered every alternate day for 4 weeks). According to the Chinese Dietary Reference Intakes, the recommended daily LYC supplementation for Chinese adults was 15 mg [29]. Accordingly, the participants in the HBO+LYC group were instructed to orally ingest three competitor brand LYC softgel capsules (5 mg LYC per capsule; the registration certificate number of domestic health food was G20150764) daily with their dinner (Figure 2).

##### Placebo-Intervention Protocol

To control for any psychological confounding factors, subjects in the HBO group received three placebo capsules daily (identical in appearance to the LYC softgel capsule) alongside HBO therapy. The CON group followed natural recovery protocols (sit quietly) combined with compressed air inhalation (composition: 21% oxygen, 78% nitrogen, and 1% other gases, 60 min/session, administered every alternate day for 4 weeks) and ingested three placebo capsules daily. No additional interventions were adopted for the CON group (Figure 2).

##### Nutritional Supplements and Diet Control

(1) Throughout the trial, all participants were prohibited from consuming any antioxidants, metabolism-enhancing substances, ergogenic nutritional supplements, or medications that could affect exercise safety (such as vitamin C, vitamin E, astaxanthin). The administration of other medications required prior notification and approval from the research team. (2) Throughout the study period, the participants across all groups were restricted from ingesting antioxidant-rich foods (such as tomatoes, carrots, blueberries, and nuts). Dietary intake was systematically monitored using a 24 h dietary recall methodology to ensure compliance. (3) A dedicated WeChat group was established for real-time participant supervision. Daily verification of nutritional supplement/placebo consumption was implemented through mandatory photographic documentation of intake procedures.

#### 2.2.3. Test Metrics

Venous blood samples (5 mL) were collected from the brachial vein by qualified healthcare professionals at 7:00 a.m. on the day before the start of the trial and on the day after the trial completion. The specimens were collected and processed for serum isolation within 30 min by using the MKE LC-LX-H165A (Lichen Instrument Technology Co., Ltd., Shanghai, China) benchtop high-speed centrifuge at 5000 rpm for 5 min. The processed serum aliquots were cryopreserved in a −80 °C ultra-low temperature freezer until batch analysis after the study completion.

##### Oxidative Stress Damage and Antioxidant Markers

Oxidative stress markers included MDA, superoxide dismutase (SOD), catalase (CAT), total antioxidant capacity (T-AOC), GPX3, and 8-hydroxy-2′-deoxyguanosine (8-OHdG). The MDA level was measured by using a microplate assay, the SOD and 8-OHdG level by using an enzyme-linked immunosorbent assay (ELISA), the CAT activity by using a spectrophotometric assay, the T-AOC level by using the ABTS assay, the GPX3 activity by using a colorimetric assay. All kits were obtained from the Nanjing Jiancheng Bioengineering Institute (Nanjing, China), and all assays were performed in accordance with the manufacturers’ instructions.

##### Inflammatory Biomarkers

The levels of inflammatory markers interleukin-6 (IL-6), IL-10, tumor necrosis factor (TNF), and C-reactive protein (CRP) were measured by using ELISA kits (the Nanjing Jiancheng Bioengineering Institute) in strict accordance with the manufacturer’s instructions, using the Huadong Electronics DG5033A microplate reader (East China Electronics Group Medical Equipment Co., Ltd., Nanjing, China).

##### Indicators for Evaluating Exercise-Induced Fatigue

The exercise-induced fatigue indicators are creatine kinase (CK), blood urea (BU), testosterone (T), and heart rate (HR). Of these, CK, BU, and T were measured using the Access 2 ImmunoAssay System (Beckman Coulter Commercial Enterprise Co., Ltd., Shanghai, China), a fully automated biochemical analyzer (U.S.). HR was measured using the LOX100A finger-clip pulse oximeter (LePu Intelligent Medical Devices Co., Ltd., Shenzhen, China) after the subjects woke up in the morning and before they engaged in any physical activity.

### 2.3. Statistical Analysis

SPSS 30.0 software was used to organize and analyze the statistical data, and GraphPad Prism 8.0 software was used to plot the statistical data. Data were expressed as the mean ± standard deviation ($\overset{-}{x}$ ± s). The Munchly test was performed to assess sphericity; when the sphericity assumption was not met, the Greenhouse–Geisser method was used for correction. One-way analysis of variance (ANOVA) was used to compare any differences in the baseline characteristics (e.g., age, height, weight, and basal metabolic rate) among the three groups. Repeated-measures ANOVA was performed to examine the main and interaction effects of oxidative stress damage and antioxidant markers, inflammatory markers, and exercise-induced fatigue assessment indicators. When interaction effects were present, simple effects were further compared. Effect sizes were calculated according to Cohen’s guidelines [30]. The *η*^2^*_p_* statistic was used to analyze effect sizes, which were classified as a trivial effect size (<0.01), small effect size (0.01–0.06), medium effect size (0.06–0.14), and large effect size (≥0.14). Statistical significance was set at *p* < 0.05.

## 3. Results

### 3.1. Pre- and Post-Intervention Variations in Oxidative Stress and Antioxidant Markers Across the Three Subject Groups

**Figure 3:**
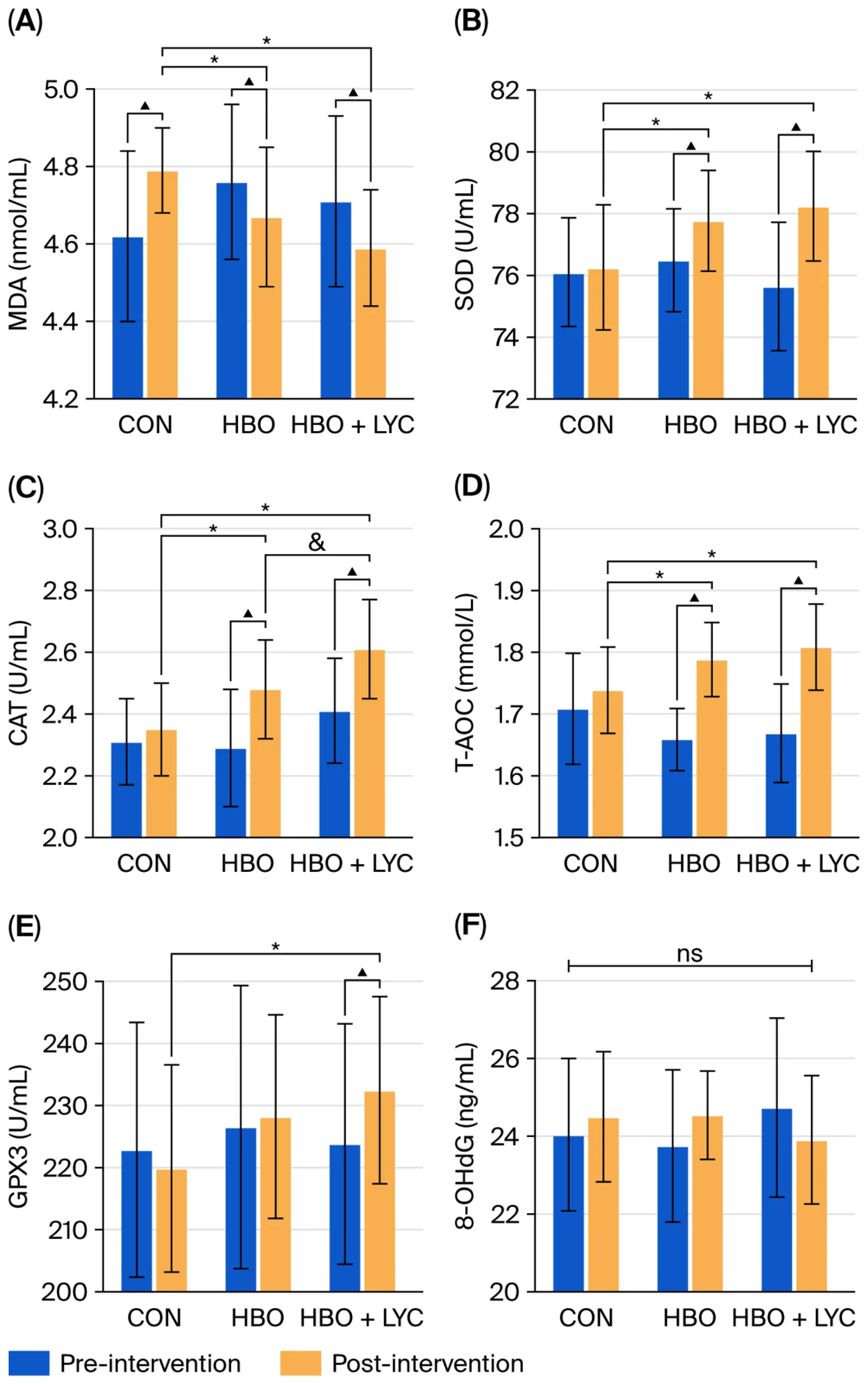
Comparisons of results within and between groups of oxidative stress and antioxidant markers after 4 weeks of intervention. (**A**) The MDA level within and between group comparisons. (**B**) The SOD activity within and between group comparisons. (**C**) CAT activity within and between group comparisons. (**D**) The T-AOC level within and between group comparisons. (**E**) The GPX3 activity within and between group comparisons. (**F**) The 8-OHdG level within and between group comparisons. The HBO and HBO+LYC groups displayed greater improvements in the levels of MDA, SOD, CAT, and T-AOC when compared to the CON group. In addition, the HBO+LYC group exhibited greater improvements in CAT and GPX3 when compared to both the HBO and CON groups. Note: ▲ significant difference after the intervention when compared to the before the intervention level, *p* < 0.05; * significant difference after the intervention when compared to the CON group, *p* < 0.05; & significant difference after the intervention when compared to the HBO group, *p* < 0.05.

As shown in Figure 3, MDA (*F* = 28.204, *p* < 0.05, *η*^2^*_p_* = 0.573); SOD (*F* = 15.521, *p* < 0.05, *η*^2^*_p_* = 0.425); CAT (*F* = 11.521, *p* < 0.05, *η*^2^*_p_* = 0.354); T-AOC (*F* = 20.977, *p* < 0.05, *η*^2^*_p_* = 0.500); and GPX3 (*F* = 8.036, *p* < 0.05, *η*^2^*_p_* = 0.277) exhibited interaction effect.

Compared with before the intervention (*pre*), MDA levels in the CON group were significantly elevated (*p* < 0.05, *η*^2^*_p_* = 0.439); compared with the CON group, MDA levels were significantly reduced in the HBO group and HBO+LYC group were significantly reduced after the intervention (*post*) (*p* < 0.05), while SOD, CAT, and T-AOC levels were significantly increased (*p* < 0.05), and GPX3 levels were significantly increased in the HBO+LYC group (*p* < 0.05). Furthermore, at *post*, compared with the HBO group, the CAT levels was significantly higher after the intervention in the HBO+LYC group (*p* < 0.05) (Table S1).

Before starting the trial, schedules for training and intervention sessions were communicated to the participants in advance. Throughout the study duration, the participants maintained good compliance with no instances of attrition, and the final sample size was set to 15 subjects per group (Figure 1).

### 3.2. Pre- and Post-Intervention Variations in Inflammatory Marker Levels Across the Three Subject Groups

**Figure 4:**
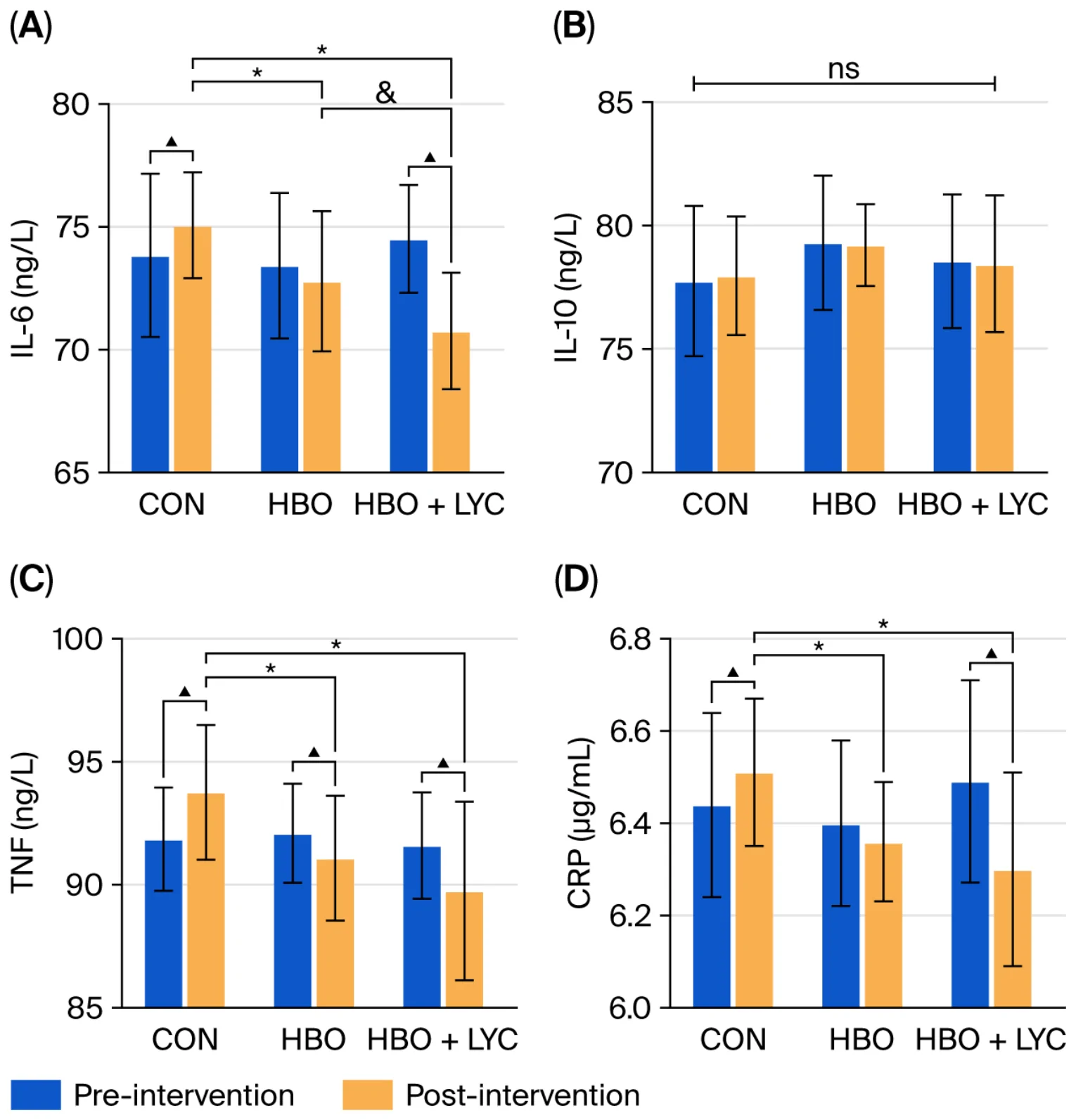
Comparisons of results within and between groups of inflammatory markers after 4 weeks of intervention. (**A**) The IL-6 level within and between group comparisons. (**B**) The IL-10 level within and between group comparisons. (**C**) The TNF level within and between group comparisons. (**D**) The CRP level within and between group comparisons. The HBO and HBO+LYC groups displayed greater improvements in the levels of CK, IL-6, TNF, and CRP when compared to those in the CON group. In addition, the HBO+LYC group exhibited greater improvements in the levels of IL-6 when compared to those in both the HBO and CON groups. Note: ▲ significant difference after the intervention when compared to the before the intervention level, *p* < 0.05; * significant difference after the intervention when compared to the CON group, *p* < 0.05; & significant difference after the intervention when compared to the HBO group, *p* < 0.05.

As shown in Figure 4, IL-6 (F = 28.301, *p* < 0.05, *η*^2^*_p_* = 0.574); TNF (*F* = 16.441, *p* < 0.05, *η*^2^*_p_* = 0.439); CRP (*F* = 23.232, *p* < 0.05, *η*^2^*_p_* = 0.525) exhibited interaction effect.

Compared with *pre*, the CON group showed significant elevated in IL-6 (*p* < 0.05, *η*^2^*_p_* = 0.137), TNF (*p* < 0.05, *η*^2^*_p_* = 0.268), and CRP (*p* < 0.05, *η*^2^*_p_* = 0.148); at *post*, compared with the CON group, IL-6, TNF, and CRP levels were significantly reduced in the HBO group and the HBO+LYC group (*p* < 0.05). Furthermore, at *post*, compared with the HBO group, the IL-6 levels was significantly reduced in the HBO+LYC group (*p* < 0.05) (Table S2).

### 3.3. Pre- and Post-Intervention Variations in Indicators for Evaluating Exercise-Induced Fatigue Levels Across the Three Subject Groups

**Figure 5:**
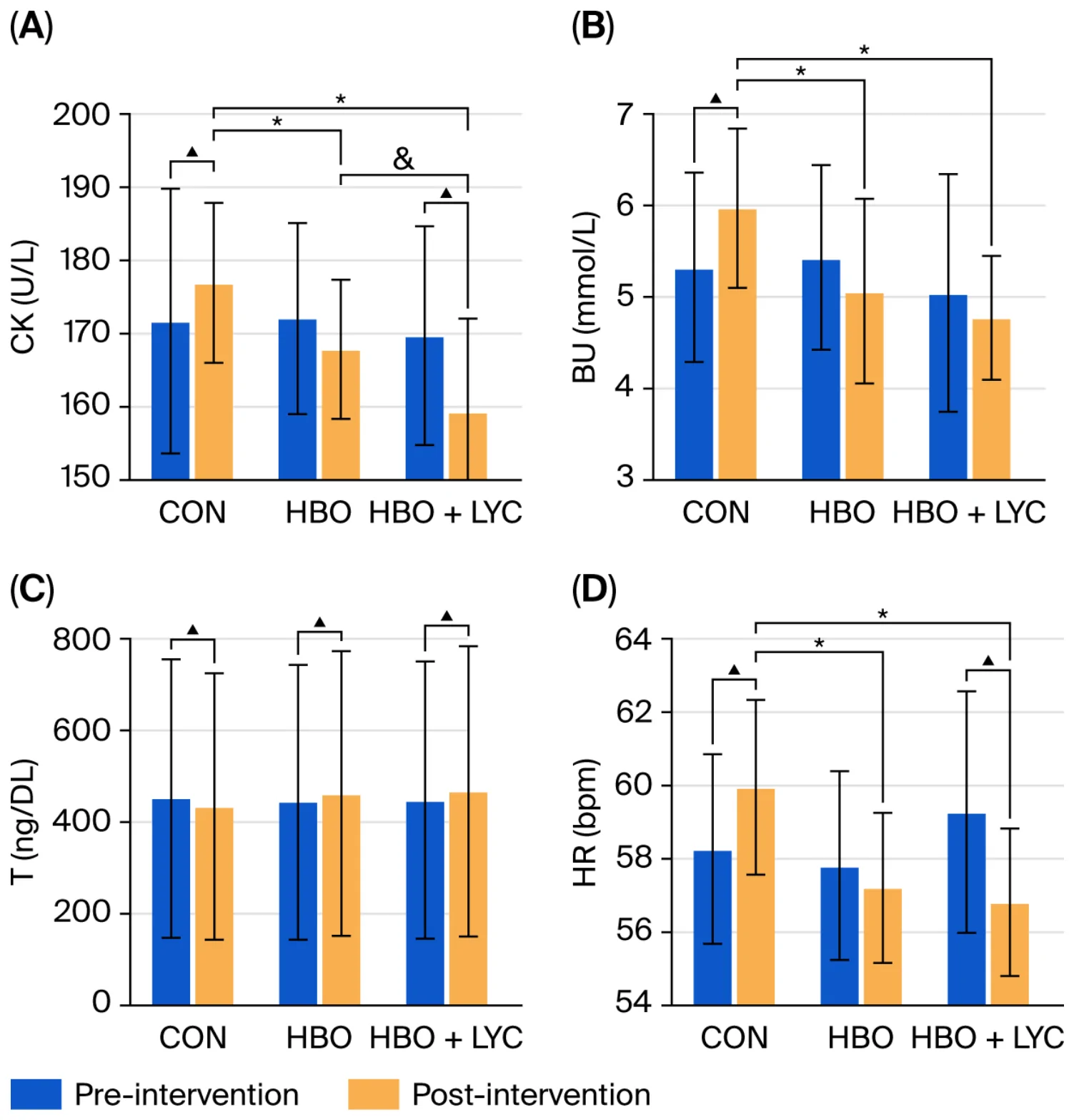
Comparisons of results within and between groups of indicators for evaluating exercise-induced fatigue after 4 weeks of intervention. (**A**) CK level within and between group comparisons. (**B**) The BU level within and between group comparisons. (**C**) The T level within and between group comparisons. (**D**) The HR level within and between group comparisons. The HBO and HBO+LYC groups displayed greater improvements in the levels of CK, BU, T, and HR when compared to those in the CON group. In addition, the HBO+LYC group exhibited greater improvements in the levels of CK when compared to those in both the HBO and CON groups. Note: ▲ significant difference after the intervention when compared to the before the intervention level, *p* < 0.05; * significant difference after the intervention when compared to the CON group, *p* < 0.05; & significant difference after the intervention when compared to the HBO group, *p* < 0.05.

As shown in Figure 5, CK (*F* = 12.107, *p <* 0.05, *η*^2^*_p_*=0.366); BU (*F* = 4.209, *p* < 0.05, *η*^2^*_p_* = 0.167); T (*F* = 7.290, *p* < 0.05, *η*^2^*_p_* = 0.258); HR (*F* = 8.314, *p* < 0.05, *η*^2^*_p_* = 0.284) exhibited an interaction effect.

Compared with *pre*, CK (*p* < 0.05, *η*^2^*_p_* = 0.112), BU (*p* < 0.05, *η*^2^*_p_* = 0.120), and HR (*p* < 0.05, *η*^2^*_p_* = 0.114) in the CON group were significantly elevated, while T (*p* < 0.05, *η*^2^*_p_* = 0.108) was significantly reduced; at *post*, compared with the CON group, CK, BU, and HR were significantly reduced in the HBO group and the HBO+LYC group (*p* < 0.05). Furthermore, at *post*, compared with the HBO group, the CK levels were significantly reduced in the HBO+LYC group (*p* < 0.05) (Table S3).

## 4. Discussion

This study demonstrated that a 4-week high-intensity badminton training program led to excessive production of ROS in athletes, disrupting the oxidative–antioxidative balance, inducing significant oxidative stress, and triggering inflammatory responses. However, a 4-week intervention with either HBO therapy alone or HBO therapy combined with LYC showed that both approaches effectively scavenged free radicals, enhanced antioxidant capacity, and reduced exercise-induced oxidative stress and inflammation. Notably, the combination of HBO and LYC produced superior therapeutic effects compared with HBO alone. To our knowledge, this is the first study to evaluate HBO or HBO+LYC in badminton players. Our research findings offer a scientifically sound and effective method for reducing oxidative stress and inflammatory responses following high-intensity badminton training and offer a theoretical basis for promoting the recovery of badminton athletes from exercise-induced fatigue.

### 4.1. Improvement of HBO+LYC Therapy on Oxidative Stress and Production of Antioxidant Biomarkers

In the present study, HBO was administered at 1.3 ATA with an oxygen concentration of 26–28%. In recent years, oxygen therapy has been increasingly used to support recovery during sports training. Low-pressure HBO, generally administered at approximately 1.3 ATA, has consequently emerged as a novel recovery method in sport [16,18,31,32,33,34]. Although the pressure and oxygen concentration of 1.3 ATA HBO therapy are relatively lower compared to medical HBO (pressure greater than 2 ATA, oxygen concentration of 100%), 1.3-ATA HBO still significantly increases dissolved oxygen content and oxygen partial pressure in the blood [16]. This physiological response can inhibit free radical production [15]. Several studies have shown that 1.3 ATA HBO can reduce oxidative stress and enhance the body’s antioxidant capacity [15,16]. In addition, 1.3 ATA HBO can reduce the risk of oxygen toxicity in athletes and is a safe and effective method for alleviating fatigue [15]. Oxygen toxicity is a common adverse event associated with HBO treatment. Although increased blood oxygen partial pressure can alleviate hypoxia in athletes, excessively high levels may induce oxygen toxicity and disrupt central nervous system function. Symptoms of oxygen toxicity include nausea, seizures, anxiety, confusion, and dizziness. Because 1.3 ATA HBO uses relatively low pressure and oxygen concentrations, it reduces the risk of oxygen toxicity in athletes. LYC, as a potent antioxidant, can likewise effectively reduce oxidative stress [21]. According to the Chinese Dietary Reference Intakes, a daily LYC dose of 15 mg can effectively reduce oxidative stress without causing lycopenemia from excessive intake [29]. Therefore, both the functional rationale and the selected dose of LYC in this study were supported by theoretical and scientific evidence. In addition, HBO and LYC share similar antioxidant mechanisms. Both interventions can reduce oxidative stress through the Nuclear factor E2-related factor 2 (NEF2L2) signaling pathway [35,36]. We therefore hypothesized that their combined use might produce a synergistic effect.

The results of this study showed that, after the intervention, the CON group exhibited a significant increase in MDA levels, accompanied by moderate elevations in T-AOC levels as well as SOD and CAT activities. This response reflects the body’s compensatory antioxidant mechanism: when oxidative stress becomes excessive, the enzymatic antioxidant system increases its activity to counteract the imbalance. Such adaptations in antioxidant enzyme activity help the body manage training-induced stress and partially restore the oxidative–antioxidant equilibrium [37]. When the body’s antioxidant defenses are insufficient to neutralize free radicals, the imbalance between oxidative and antioxidant systems results in oxidative stress and subsequent cellular damage. This metabolic disturbance can impair energy metabolism and disrupt normal muscular physiological processes [38,39]. One key factor contributing to the heightened oxidative stress response after high-intensity exercise is cellular hypoxia. HBO therapy helps alleviate this condition by enhancing oxygen availability, thereby reducing exercise-induced oxidative stress.

In this study, post-intervention results showed that the HBO group experienced a significant reduction in MDA levels, accompanied by notable increases in T-AOC, SOD, and CAT activities. These outcomes suggest that HBO therapy effectively eliminates ROS generated during high-intensity exercise in athletes and enhances overall antioxidant capacity. These findings align with previous research by Qu et al. [15], who reported that six HBO sessions (once daily at 1.25 ATA for 60 min/session) significantly reduced MDA levels and increased SOD activity in participants. Our research group has also provided supporting evidence: a four-week HBO therapy regimen (1.3 ATA, 60 min/session, every other day) significantly lowered MDA and protein carbonyl levels while enhancing T-AOC, SOD, and CAT activities in athletes during summer training [16]. Taken together, these findings demonstrate that repeated HBO therapy at 1.25–1.3 ATA effectively reduces oxidative stress induced by high-intensity exercise and enhances systemic antioxidant capacity. HBO therapy reduces oxidative stress in the body after high-intensity exercise, primarily through cellular hypoxia-induced impairment of energy metabolism. Cytochrome oxidase does not reduce oxygen into water, causing the accumulation of excess oxygen in the extracellular space and the cytoplasm, which cannot be utilized by the mitochondria. These excessive oxygen atoms then capture electrons from the surrounding molecules to subsequently form reactive oxygen-free radicals [40]. HBO therapy can improve cellular hypoxia by increasing the physically dissolved oxygen content in the blood, thereby reducing the generation of free radicals. In addition, HBO therapy improves oxygen partial pressure, thereby activating the transcription factors and gene expression, ultimately increasing the antioxidant enzyme activity and reducing oxidative stress [40]. During this process, the expression of proteins involved in the kelch-like ECH-associated protein-1 (KEAP1)/NFE2-like bZIP transcription factor 2 (NFE2L2)/heme oxygenase 1 (HMOX1)-signaling pathway plays an important role [41]. Under normal physiological conditions, NFE2L2 forms a dimer with the receptor KEAP1 in the cytoplasm and remains at a low level through the action of the ubiquitin–proteasome system. With increasing levels of oxidative stress, NFE2L2 dissociates from KEAP1 and is translocated into the nucleus, where it recognizes and binds to the antioxidant response element (ARE). In addition, the NFE2L2-signaling pathway mediates cellular defense mechanisms through the transcriptional regulation of several known target genes, including HMOX1. These NFE2L2-dependent enzymes then effectively reduce the free radical burden by enhancing the antioxidant defense systems [42]. However, when the increase in the antioxidant enzyme activity is relatively low, oxidative stress damage may occur in the body. HBO therapy can reduce the expression of KEAP1, triggering further release of NFE2L2, which enhances HMOX1 expression, a key downstream factor of the NFE2L2 protein. HMOX1 can catalyze the oxidative metabolism of heme to produce nitric oxide, thereby protecting the cells from oxidative stress damage. Meanwhile, HBO therapy can increase the partial pressure of oxygen, promote the translocation of NFE2L2 to the nucleus and binding to ARE, to form the NFE2L2-ARE-signaling pathway, and induce the transcription of downstream genes for antioxidant enzymes and antioxidants [14]. In addition, the increased oxygen partial pressure will induce hypoxia-inducible factor 1 subunit alpha (HIF1A). HIF1A can translocate to the nucleus and retain its stability there to form a heterodimer with ARNT, followed by activation of the expression of downstream genes, thereby reducing both cellular oxygen consumption and reactive oxygen species production.

The results of this study revealed that HBO therapy alone did not significantly influence GPX3 activity, a finding consistent with the observations of Benedetti et al. [43]. A possible explanation is that HBO does not directly affect the specific substrates (e.g., reduced glutathione) or the enzymatic conditions necessary for GPX3 synthesis. In contrast, the combined intervention group exhibited a significant increase in GPX3 levels, suggesting that LYC supplementation supports the substrates and enzymatic environments required for GPX3 production. This outcome aligns with the findings of Ramaswamy et al. [44], who reported that supplementing male track and field athletes with tomato juice (10 mg LYC/day for 60 days) significantly decreased thiobarbituric acid reactive substances following morning training, while simultaneously elevating GPX3 levels. Consistent with the findings of Kawada et al. [45], this study showed that neither HBO nor HBO+LYC interventions significantly affected 8-OHdG levels. Several factors may explain this outcome. First, DNA oxidative damage is strongly influenced by exercise intensity, particularly high-intensity strength training. Since the athletes in this study did not perform daily high-intensity strength training, they likely avoided severe muscle fiber strain and DNA damage. Second, all participants were young athletes with extensive long-term training backgrounds. As a result, their bodies had developed adaptive mechanisms to cope with high-intensity exercise, including highly efficient DNA repair systems. These adaptations likely enabled them to repair oxidative DNA damage rapidly, thereby minimizing detectable DNA injury after the intervention. Hence, the 8-OHdG activity of the participants in this study did not increase, indicating that HBO therapy and combined interventions could not further reduce the 8-OHdG levels.

This study further demonstrated that the HBO+LYC group achieved greater improvements than the HBO-only group, with more pronounced reductions in the MDA levels and enhanced increases in T-AOC, SOD, and CAT activities. Based on existing evidence, the superior effects of HBO+LYC may be attributed to additional reduction in exercise-induced oxidative stress conferred by LYC beyond that achieved with HBO therapy alone. Supporting this finding, Kim et al. found that an 8-week supplementation with LYC (15 mg/day) reduced DNA damage in lymphocytes from healthy men, increased the plasma SOD activity, and protected endothelial function through anti-inflammatory effects [46]. Guo et al. also reached similar conclusions, finding that LYC activates the NEF2L2/HO-1/SIRT1 pathway, reduces reactive oxygen species (ROS), inflammatory factors, cell adhesion, and apoptosis rates under oxidative stress conditions, and alleviates hydrogen peroxide-induced oxidative damage in human vascular endothelial cells [47]. Thus, the combination of HBO therapy with LYC may provide enhanced protection against oxidative stress following high-intensity exercise. As a potent antioxidant, LYC is a key component of the non-enzymatic antioxidant defense system. Compared to other carotenoids and vitamin E, it possesses superior free radical-scavenging capacity. LYC can neutralize peroxide-derived free radicals through physical quenching mechanisms and activate the NEF2L2–ARE-signaling pathway, which enhances antioxidant enzyme activity and reduces oxidative stress [48,49]. These findings indicate that LYC supplementation, when combined with HBO therapy, may further eliminate free radicals and strengthen the body’s antioxidant defense.

### 4.2. Improvement of HBO+LYC Therapy on Inflammatory Biomarkers

During exercise-induced fatigue, oxidative stress and immune suppression can trigger inflammatory reactions, making inflammatory markers valuable indicators for monitoring the onset of fatigue in athletes. In this study, the CON group exhibited significant increases in IL-6, TNF, and CRP levels, demonstrating that high-intensity exercise stimulates a substantial release of pro-inflammatory cytokines and amplifies the body’s inflammatory response. These results are consistent with previous research findings. Chronic, persistent inflammation has been shown to delay muscle recovery, increase the risk of sports-related injuries, weaken the immune system’s ability to fight viral infections, and ultimately impair athletic performance [50,51]. Oxidative stress plays a central role in the development of inflammation [52]. After high-intensity exercise, excessive ROS accumulation not only damages membrane lipids, leading to structural and functional impairments at the cellular level, but also stimulates the release of pro-inflammatory cytokines, chemokines, and other signaling molecules that exacerbate the inflammatory response.

In this study, the HBO group exhibited significant reductions in IL-6, TNF, and CRP levels, indicating that HBO therapy effectively alleviates inflammatory responses in athletes following high-intensity exercise. This result aligns with previous research on the anti-inflammatory effects of HBO. For instance, Qu et al. reported that six sessions of HBO therapy (1.25 ATA, 60 min/session, once daily) after exhaustive exercise significantly reduced CK levels in participants [15]. Similarly, Zhu found that a four-week HBO intervention (1.3 ATA, 60 min/session, every other day) conducted during both winter and summer training significantly decreased CK, IL-6, TNF, and CRP levels in skeletal athletes [16]. The anti-inflammatory effects of HBO therapy following high-intensity exercise are largely attributed to its ability to scavenge exercise-induced ROS and enhance systemic antioxidant defenses, thereby reducing inflammatory responses [53,54,55]. However, de Wolde et al. reported no significant changes in plasma levels of MDA, TNF, IL-6, IL-8, or IL-10 in healthy subjects after only three HBO sessions [56]. A key distinction is that the intervention in their study involved a substantially lower treatment dosage (three sessions) compared with other research protocols, which typically consist of 6–18 sessions. This insufficient exposure likely failed to reach the threshold necessary to produce measurable anti-inflammatory effects. Moreover, the participants in that study were healthy individuals without notable systemic inflammation. Consequently, their baseline levels of inflammatory markers were much lower than those typically observed in athletes, leaving little room for HBO therapy to induce further reductions. Taken together, current evidence suggests that short-term or low-dose HBO interventions have limited anti-inflammatory effects. Detectable changes in inflammatory biomarkers appear to require a minimum cumulative treatment dose sufficient to trigger dose-dependent physiological adaptations.

Our study further revealed that the HBO+LYC group experienced greater reductions in IL-6, TNF, and CRP levels compared with the HBO-only group. Based on existing evidence, the superior effects of HBO+LYC may be attributed to the additional reduction in exercise-induced inflammation conferred by LYC beyond that achieved with HBO therapy alone. Supporting this finding, Taherireykande et al. reported that participants who consumed tomato juice (50 mg of LYC per day) for one week and subsequently performed exhaustive exercise experienced a significant decrease in the blood CRP levels [57]. Similarly, Tsitsimpikou et al. demonstrated that replacing carbohydrate-rich beverages with tomato juice (2.79 ± 0.211 mg LYC/100 mL) over a 2-month training period led to significant reductions in the serum CK, CRP, and homocysteine levels in athletes [58]. The combination of HBO and LYC may provide additional protection against high-intensity exercise-induced inflammation. This effect is likely related to the antioxidant properties of LYC, which help neutralize free radicals generated during exercise, enhance overall antioxidant capacity, and thereby reduce inflammatory responses. However, in this study, IL-10 levels did not change significantly in either the HBO or HBO+LYC groups. As an anti-inflammatory cytokine, IL-10 plays a key role in suppressing macrophage-mediated release of pro-inflammatory factors and maintaining the balance between pro- and anti-inflammatory responses, ultimately contributing to the regulation of inflammation [59]. In this study, no significant changes in IL-10 levels were observed, which may be explained by several factors. First, inflammatory responses in athletes are influenced by exercise intensity and training volume. Prolonged training can induce a marked inflammatory response, and the increases in inflammatory markers seen in the CON group may reflect the cumulative effects of four weeks of training. Second, previous studies suggest that compared with pro-inflammatory cytokines, the expression and production of anti-inflammatory factors, such as IL-10, occur more slowly and require a longer activation period. Therefore, the absence of a significant increase in IL-10 levels in this study may be due to insufficient activation time for its production pathway. Second, pro-inflammatory factors appeared highly responsive to both HBO and HBO+LYC interventions, demonstrating clear reductions. In contrast, IL-10 showed relatively low sensitivity to these treatments, which may explain its lack of adaptive changes following the interventions. HBO and HBO+LYC may reduce inflammation by modulating other anti-inflammatory cytokines rather than IL-10. To explore this mechanism, future studies will examine additional anti-inflammatory factors, such as IL-2 and IL-4, to better understand the effects of HBO therapy and the combined intervention.

Oxidative stress damage caused by high-intensity exercise is one of the key biological mechanisms underlying exercise-induced fatigue. Moderate-to-low-intensity training induces a moderate oxidative stress response in the body, activating the antioxidant enzyme system, enhancing cellular antioxidant capacity, and maintaining homeostasis by participating in the regulation of energy metabolism, cellular signal transduction, and gene expression, thereby inducing adaptive changes in cells [60]. However, excessive ROS generated during high-intensity exercise can directly attack the complexes of the mitochondrial respiratory chain, causing structural damage and reducing electron transport efficiency. At the same time, this event promotes mitochondrial membrane lipid peroxidation, disrupts cell membrane integrity, and inhibits adenosine triphosphate synthesis, impairing the cellular energy supply [61]. Furthermore, excessive ROS can compromise the integrity of the blood–brain barrier, increasing its permeability and facilitating the entry of peripheral pro-inflammatory factors and ROS into the brain, thereby activating microglia, triggering a neuroinflammatory response, and disrupting the survival environment of central nervous system cells. This chain of events leads to multidimensional regulatory disorders within the central nervous system, including neurotransmitter levels, energy metabolism, and ionic balance, thereby inhibiting the central nervous system’s ability to drive and regulate movement [62,63]. Therefore, suppressing oxidative stress and inflammatory responses following high-intensity exercise can effectively promote the recovery from exercise-induced fatigue.

To assess changes in athletes’ fatigue status following the trial, this study further examined changes in indicators such as CK, BU, T, and HR. Multiple studies have confirmed that CK, BU, T, and HR are effective indicators for monitoring the onset of exercise-induced fatigue; these indicators can effectively evaluate changes in physical performance following high-intensity exercise from perspectives such as cellular metabolism [64,65,66,67]. This study found that post-intervention levels of CK, BU, T, and HR exhibited significant improvement in both the HBO alone and HBO+LYC groups, indicating alleviation of the athletes’ fatigue. Therefore, this study suggests that both HBO and LYC promote the recovery from exercise-induced fatigue by suppressing oxidative stress and inflammatory responses. However, because athletes differ substantially at the individual level, additional evidence is needed to support this conclusion. Although all participants followed the same training program, they differed in physiological function and exercise capacity. These differences may have resulted in varying degrees of hypoxia during training and, consequently, different levels of oxidative stress and inflammatory responses. The effects of HBO may therefore vary among individual athletes. In addition, athletes may also differ in their ability to absorb LYC. Even when the same dose is administered, the amount ultimately absorbed and utilized by cells may vary between individuals. Such interindividual variability may be more pronounced between male and female athletes and may limit the generalizability of our findings. Future studies will therefore measure cellular oxygen levels and blood LYC concentrations in individual athletes. These measurements will help determine interindividual differences in responses to HBO and LYC interventions.

## 5. Limitations of This Study

This study focused only on oxidative stress and inflammatory markers without exploring the molecular mechanisms underlying the observed changes. In addition, measurements were taken only on the day following the intervention, which limits our ability to assess how long the effects of HBO and HBO+LYC on these indicators persist. Therefore, the mechanism by which the intervention influences long-term adaptations is unclear. furthermore, the potential side effects of prolonged HBO or HBO+LYC treatment in athletes were not evaluated, leaving questions about the safety of extended use.

This study included 45 badminton players as participants; however, this sample size may still be insufficient and could limit the generalizability of the findings.

Although this study ensured that the gender ratios were consistent across the three groups, which helps avoid the influence of gender on the effects between groups, we did not determine whether athletes of different genders within the same group exhibit differences in their responsiveness to the same intervention.

## 6. Conclusions and Recommendations

### 6.1. Conclusions

Four weeks of high-intensity badminton training led to a marked increase in free radical production, resulting in oxidative stress and subsequent inflammatory responses in athletes. Interventions with either HBO alone or in combination with LYC effectively reduced the MDA, IL-6, TNF, CRP, CK, BU, and HR levels, while increasing the activities of SOD and CAT as well as the levels of T-AOC and T. Moreover, the combined HBO+LYC intervention proved more effective than HBO alone for certain outcomes, including enhanced GPX3 activity. Thus, we recommend that athletes use HBO and HBO+LYC interventions to reduce oxidative stress and inflammatory response so as to accelerate recovery from exercise-induced fatigue after high-intensity exercise.

### 6.2. Recommendation

(1) Future studies should extend the intervention period and examine whether the effects exhibit a positive time- and dose–response relationship. They should also determine whether these effects can be maintained over the long term.

(2) Future studies should recruit larger samples and include athletes from a broader range of sports. This would improve the generalizability and practical applicability of the findings.

(3) Future studies should examine whether male and female athletes differ in their responsiveness to HBO and HBO+LYC interventions. The results could support the development of sex-specific intervention protocols for athletes.

## Figures and Tables

**Table 1:** Fundamental information of the subjects ($\bar{x}$ ± s, *n* = 45).

Index	CON	HBO	HBO+LYC	*F*	*p*	*η* ^2^ *_p_*
Age (year)	20.07 ± 1.10	19.93 ± 0.79	20.47 ± 1.06	1.167	0.321	0.053
Height (cm)	169.93 ± 8.50	172.33 ± 7.31	171.87 ± 7.15	0.412	0.665	0.019
Weight (kg)	63.81 ± 12.63	61.33 ± 8.32	66.42 ± 9.09	0.937	0.400	0.043
BMI (weight/height^2^)	21.91 ± 2.99	20.54 ± 1.59	22.45 ± 2.41	2.511	0.093	0.107
Skeletal Muscle Mass (kg)	29.57 ± 6.35	29.80 ± 5.58	30.34 ± 5.87	0.067	0.935	0.003
Percentage Body Fat (%)	16.66 ± 5.42	13.88 ± 6.37	18.41 ± 6.21	2.163	0.128	0.093
Basal Metabolic Rate (kcal)	1487.46 ± 233.23	1474.33 ± 198.58	1578.13 ± 223.72	0.999	0.377	0.045

**Table 2:** Weekly training program for the three subject groups.

Time		Training Content	Exercise Intensity and Duration
Monday to Saturday		Warm-up: 15 min of uniformly programmed warm-up session for all the following training sessions: general aerobic 5 min; dynamic stretching activation 10 min	Exercise intensity: (1) 60–70% HRmax, (2) RPE: 4–5 points Exercise duration: 15 min
1–4 weeks	Monday, Wednesday	Specialized physical fitness training × 2 h; Specialized technical training × 2 h	Exercise intensity: (1) 75–90% HRmax; (2) Blood lactate: 12–14 mmol/L; (3) RPE: 5–8 Exercise duration: Total of 210 min (105 min in the afternoon + 105 min in the evening)
	Tuesday, Thursday. Friday, Saturday	Specialized technical training × 2 h	Exercise intensity: RPE: 5–7 Exercise duration: 105 min
	Sunday	Rest day	

## Data Availability

The original contributions presented in this study are included in the article/Supplementary Materials. Further inquiries can be directed to the corresponding author.

## Supplementary Materials

The following supporting information can be downloaded at: https://www.mdpi.com/article/10.3390/nu18172861/s1, Table S1. Comparisons of results within and between groups of oxidative stress and production of antioxidant biomarkers after 4 weeks of intervention ($\overset{-}{x}$ ± s, *n* = 45). Table S2. Comparisons of results within and between groups of Inflammatory marker after 4 weeks of intervention ($\overset{-}{x}$ ± s, *n* = 45). Table S3. Comparisons of results within and between groups of Evaluating Exercise-Induced Fatigue Levels after 4 weeks of intervention ($\overset{-}{x}$ ± s, *n* = 45).
