# Supplementary material for: Effects of Hyperbaric Oxygen Combined with Lycopene on Oxidative Stress and Inflammation in Collegiate Badminton Players After High-Intensity Training
Source: Nutrients. 2026 Sep 2;18(17):2861. doi: 10.3390/nu18172861 (PMC13567025; doi:10.3390/nu18172861)
Supplement: Supplementary file 1 [file nutrients-18-02861-s001.zip › nutrients-4442135-supplementary.pdf]

Table S1. Comparisons of results within and between groups of oxidative stress and production of antioxidant biomarkers after 4 weeks of intervention (x̄±s, n=45).

Index	Group	Pre-intervention	Post-intervention	F	p	η^2_p
	CON	4.62±0.22	4.79±0.11▲			
MDA（nmol/ml）	HBO	4.76±0.20	4.67±0.18▲*	28.204	<0.05	0.573
	HBO+LYC	4.71±0.22	4.59±0.15▲*			
	CON	76.10±1.76	76.26±2.03			
SOD（U/ml）	HBO	76.49±1.67	77.77±1.64▲*	15.521	< 0.05	0.425
	HBO+LYC	75.64±2.08	78.24±1.77▲*			
	CON	2.31±0.14	2.35±0.15			
CAT（U/ml）	HBO	2.29±0.19	2.48±0.16▲*	11.521	< 0.05	0.354
	HBO+LYC	2.41±0.17	2.61±0.16▲*&			
	CON	1.71±0.09	1.74±0.07			
T-AOC（mmol/L）	HBO	1.66±0.05	1.79±0.06▲*	20.977	< 0.05	0.500
	HBO+LYC	1.67±0.08	1.81±0.07▲*			
	CON	222.88±20.55	219.88±16.72			
GPX3（U/ml）	HBO	226.53±22.84	228.18±16.42	8.036	< 0.05	0.277
	HBO+LYC	223.81±19.39	232.45±15.09▲*			
	CON	24.04±1.96	24.50±1.68			
8-OHdG（ng/ml）	HBO	23.75±1.96	24.54±1.14	2.524	> 0.05	0.107
	HBO+LYC	24.74±2.30	23.91±1.65			

Note: ▲significant difference after the intervention when compared to the before the intervention level, $p < 0.05$; * significant difference after the intervention when compared to the CON group, $p < 0.05$; & significant difference after the intervention when compared to the HBO group, $p < 0.05$ (the same as below).

Table S2. Comparisons of results within and between groups of Inflammatory marker after 4 weeks of intervention ($\bar{x}$±s, n=45)

Index	Group	Pre-intervention	Post-intervention	F	p	η^2_p
IL-6（ng/L）	CON	73.84±3.33	75.06±2.17▲	28.301	< 0.05	0.574
	HBO	73.42±2.97	72.78±2.87*			
	HBO+LYC	74.51±2.20	70.76±2.38▲*&			
IL-10（ng/L）	CON	77.74±3.06	77.96±2.41	0.095	> 0.05	0.005
	HBO	79.30±2.73	79.20±1.67			
	HBO+LYC	78.55±2.71	78.44±2.78			
TNF（ng/L）	CON	91.87±2.09	93.77±2.74▲	16.441	< 0.05	0.439
	HBO	92.10±2.01	91.09±2.53▲*			
	HBO+LYC	91.61±2.16	89.76±3.63▲*			
CRP（μg/ml）	CON	6.44±0.20	6.51±0.16▲	23.232	< 0.05	0.525
	HBO	6.40±0.18	6.36±0.13*			
	HBO+LYC	6.49±0.22	6.30±0.21▲*			

Table S3. Comparisons of results within and between groups of Evaluating Exercise-Induced Fatigue Levels after 4 weeks of intervention ($\bar{x}$±s, n=45)

Index	Group	Pre-intervention	Post-intervention	F	p	η^2_p
	CON	171.73±18.05	176.93±10.95▲			
CK（U/L）	HBO	172.06±13.07	167.86±9.53*	12.107	< 0.05	0.366
	HBO+LYC	169.73±14.96	159.33±12.73▲*&			
	CON	5.32±1.04	5.97±0.87▲			
BU（mmol/L）	HBO	5.43±1.01	5.06±1.01*	4.209	< 0.05	0.167
	HBO+LYC	5.04±1.30	4.77±0.68*			
	CON	452.24+302.58	434.83+290.13▲			
T（ng/DL）	HBO	444.18+298.89	462.30+310.44▲	7.290	< 0.05	0.258
	HBO+LYC	448.58+301.86	467.66+316.52▲			
	CON	58.26±2.57	59.93±2.37▲			
HR（bpm）	HBO	57.80±2.56	57.20±2.04*	8.314	< 0.05	0.284
	HBO+LYC	59.26±3.28	56.80±2.00▲*			
